# Complete genome sequence of strain *Lactiplantibacillus plantarum* beLP1 a potential probiotic strain with antimicrobial properties isolated from kimchi

**DOI:** 10.1128/mra.00339-24

**Published:** 2024-06-11

**Authors:** Kwon Il Han, Hyun-Dong Shin, Yura Lee, Sunhwa Baek, Eunjung Moon, Youn Bum Park, Junhui Cho, Jin-Ho Lee, Ranjith Kumar Manoharan

**Affiliations:** 1 Research and Development Center, Bereum Co., Ltd., Wonju, South Korea; 2 Division of Biological Science and Technology, Yonsei University, Wonju, South Korea; The University of Arizona, Tucson, Arizona, USA

**Keywords:** *Lactiplantibacillus plantarum*, probiotic, kimchi, whole-genome sequencing

## Abstract

The complete genome of the potential probiotic *Lactiplantibacillus plantarum* strain beLP1, isolated from kimchi in South Korea, was sequenced using Illumina and PacBio technologies. The genome comprises one circular chromosome and one plasmid without antimicrobial resistance genes.

## ANNOUNCEMENT

*Lactiplantibacillus plantarum* is known for its probiotic properties, including antimicrobial activity, gastrointestinal protection, and absence of virulence and antimicrobial-resistance genes. It is generally recognized as safe for human health (1). We isolated a potential strain from cabbage kimchi (*n* = 20) without anchovy ferment in Pyeongchang, South Korea, in 2020 as previously described (2) and identified as *L. plantarum* beLP1 by 16S rRNA gene sequencing (3) (Macrogen Inc., South Korea) in August 2021. This strain exhibited antibacterial activity against *Escherichia coli*, *Staphylococcus aureus*, and *Pseudomonas aeruginosa*.

A fresh beLP1 colony was grown in De Man, Rogosa and Sharpe (MRS) broth for 24 h at 37°C, and gDNA was extracted using the MagListo 5M Genomic DNA Extraction Kit (Qiagen) for long- and short-read whole-genome sequencing (WGS). The WGS was performed using PacBio Sequel System (Pacific Biosciences, USA) and Illumina platform (Illumina, USA) (4). DNA quality and quantity were confirmed with an Agilent 2100 Bioanalyzer using a DNA 1,000 chip. A total of 3 µg of gDNA was sequenced with single-molecule real-time (SMRT) technology, and a library was prepared using the SMRTbell Express kit v2.0 (PacBio) (5). SMRT sequencing produced 129,036 subreads (1,170,295,114 bases; N50: 10,829 bp; mean length: 9,069 bp), which were *de novo* assembled using the Microbial Assembly application in SMRT Link v10.2 (PacBio).

After *de novo* assembly, Illumina sequencing was used to correct errors in the PacBio sequences and construct more accurate contigs with the TruSeq Nano DNA library preparation kit (Illumina, USA). This yielded 13,311,004 quality-filtered 2 × 151 bp paired-end reads (≥90% of bases with Phred scores >30). After assembly, the Illumina short-read reads were subjected to error correction and adapter trimming with Trimmomatic v0.38 with default parameters (6). A total of 3,190,516 bp (N50, 3,179,857; mean length, 1,595,258) was yielded after paired-end data were then mapped against the PacBio assembly using BWA-MEM v0.7.17 and error correction using Pilon v1.21 (7). As a result of oriC Rotation, one circular chromosome and one plasmid were generated (Table 1).

**TABLE 1:** Whole-genome sequencing overview of beLP1 strain

Genetic element	Size (bp)	GC content (%)	No. of coding genes	No. of rRNAs	No. of tRNAs	Sequencing depth	GenBank accession no.
Chromosome	3,179,857	44.5	2,926	16	64	333.2	CP137847.1
Plasmid pLPRK1	10,659	36.5	11	0	0	178.8	CP137848.1
Total	3,190,516	47.4	2,937	16	64	332.6	

Genome annotation of the assembly was performed using NCBI Prokaryotic Genome Annotation Pipeline v6.2 (8). Genome annotation reported 2,937 genes, which include 2,926 coding genes and 80 RNA genes. The average nucleotide identity analysis (9) revealed that beLP1 had 99.8% sequence similarity with *L. plantarum* 299 v (Accession No. NZ_LEAV01000004). The ResFInder v4.1 (10) and CARD v3.2.3 (11) confirmed the absence of antimicrobial resistance genes in the beLP1 genome using default settings. Further research could explore how heat-killed beLP1 cells support human gut health through their metabolic activity.

## Data Availability

This project is available at GenBank under accession number PRJNA1032254 (GenBank, CP137847, CP137848; BioSample, SAMN37988430; SRA, SRX24541733, SRX24541732)
